# Causal associations between gut microbiota and adverse pregnancy outcomes: A two-sample Mendelian randomization study

**DOI:** 10.3389/fmicb.2022.1059281

**Published:** 2022-12-16

**Authors:** Chuang Li, Caixia Liu, Na Li

**Affiliations:** ^1^Department of Obstetrics and Gynecology, Shengjing Hospital of China Medical University, Shenyang, China; ^2^Key Laboratory of Maternal-Fetal Medicine of Liaoning Province, Shenyang, China

**Keywords:** Mendelian randomization, instrumental variable, gut microbiota, adverse pregnancy outcomes, causal relationship

## Abstract

Growing evidence indicates that gut microbiota could be closely associated with a variety of adverse pregnancy outcomes (APOs), but a causal link between gut microbiome and APOs has yet to be established. Therefore, in this study, we comprehensively investigated the relationship between gut microbiota and APOs to identify specific causal bacteria that may be associated with the development and occurrence of APOs by conducting a two-sample Mendelian randomization (MR) analysis. The microbiome genome-wide association study (GWAS) from the MiBioGen consortium was used as exposure data, and the GWAS for six common APOs was used as outcome data. Single-nucleotide polymorphisms (SNPs) that significantly correlated to exposure, data obtained from published GWAS, were selected as instrumental variables (IVs). We used the inverse variance-weighted (IVW) test as the main MR analysis to estimate the causal relationship. The Mendelian randomization pleiotropy residual sum and outlier (MR-PRESSO) and MR-Egger regression were used to confirm the presence of horizontal pleiotropy and to exclude outlier SNPs. We performed Cochran's Q test to assess the heterogeneity among SNPs associated with each bacterium. The leave-one-out sensitivity analysis was used to evaluate whether the overall estimates were affected by a single SNP. Our analysis shows a causal association between specific gut microbiota and APOs. Our findings offer novel insights into the gut microbiota-mediated development mechanism of APOs.

## Introduction

Adverse pregnancy outcomes (APOs) include all pathological pregnancy and childbirth complications. Many public health and medical interventions have been implemented to reduce APOs, yet the incidence of APOs remains high. APOs are a leading cause of maternal, fetal, and neonatal deaths; severe maternal morbidity; and maternal intensive care unit admissions (Rana et al., 2019). APOs are widely recognized as major public health problems due to short- and long-term adverse impacts on mothers and infants.

The gut microbiota is a massive complex community of microbial species living in the digestive tract that not only participates in the digestion of food and the absorption of nutrients but also is involved in physiological regulation of the host through the production of hormonally active substances (Luo et al., 2022). Increasing evidence indicates that microbiome dysbiosis might play an important role in the pathogenesis of APOs (Qi et al., 2021). Zheng et al. (2020) found that dynamics in gut microbiota during the first half of pregnancy differed significantly between gestational diabetes mellitus (GDM) and normoglycemic pregnant women. Another case–control study showed that pregnant women with pre-eclampsia (PE) had a high abundance of *Fusobacterium* and *Veillonella* and a relatively low abundance of *Faecalibacterium* and *Akkermansia* compared with normotensive pregnant women (Chen C. et al., 2020; Chen X. et al., 2020). Yin et al. (2021) reported that women with preterm birth (PTB) showed a distinct gut microbiome dysbiosis compared with those who delivered at term. Opportunistic pathogens, particularly *Porphyromonas, Streptococcus, Fusobacterium*, and *Veillonella*, were enriched, while *Coprococcus* and *Gemmiger* were markedly depleted in the preterm group (Yin et al., 2021). However, whether these associations are causal has not been established because of potential biases including residual confounding factors and reverse causality.

Mendelian randomization (MR) is an approach integrating summary data of the genome-wide association study (GWAS) and resembles a randomized controlled trial by leveraging allelic randomization during meiosis and subsequent irreversible exposure to genotype at conception (Lawlor et al., 2008; Ellervik et al., 2019; Ference et al., 2019). Using genetic variants as instrumental variables (IVs), the MR design is typically less likely to be affected by residual confounding factors and reverse causation than conventional observational analysis, thereby strengthening the causal relationship between exposure and outcomes (Chen C. et al., 2020; Chen J. et al., 2020; Yuan and Larsson, 2022).

**Figure 1:**
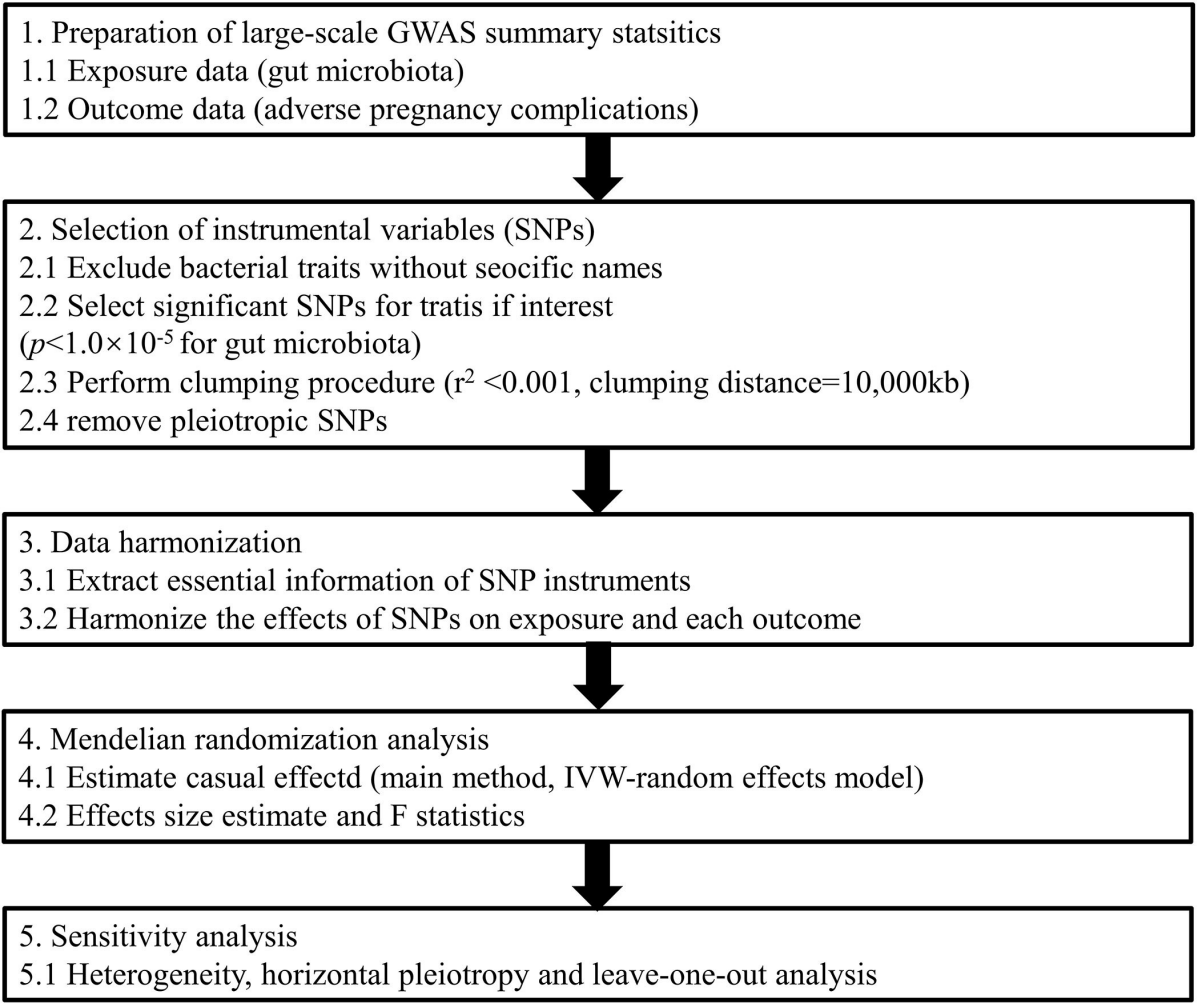
Study design of the present MR study on the associations of gut microbiota and adverse pregnancy complications. SNP, single-nucleotide polymorphism; IVW, inverse variance-weighted; MR, Mendelian randomization.

Exploring causal associations between gut microbiota and APOs not only deepens our understanding of pathogenesis derived from enteric bacteria but also facilitates the development of personalized treatments for pregnancy complications based on dietary or microbiome interventions (Di Simone et al., 2020). Elucidating the causal inference on the gut microbiome and its association with APOs is warranted. Thus, in this study, we conducted a two-sample MR analysis to evaluate the causal association between gut microbiota and six common APOs, namely, postpartum hemorrhage (PPH), abruptio placenta (AP), spontaneous abortion (SAB), premature rupture of membrane (PROM), gestational diabetes mellitus (GDM), and pre-eclampsia (PE) or eclampsia (Figure 1). To our knowledge, this is the first study assessing the causal role of gut microbiome in the development of pregnancy complications.

## Materials and methods

### Data sources

We obtained the GWAS dataset of human gut microbiome from the MiBioGen consortium (Kurilshikov et al., 2021). The MiBioGen study is the largest, multi-ethnic, and genome-wide analysis to investigate host –genetics– microbiome associations (Wang et al., 2018). This study coordinated 16S rRNA gene sequencing profiles and genotyping data of 18,340 participants from 24 population-based cohorts comprising 211 bacterial taxa (Kurilshikov et al., 2021), among which 15 microbial taxa without specific species name (unknown family or genus) were excluded, resulting in a total of 196 bacterial taxa being included in the present study.

GWAS summary data on PPH (3,670 cases and 98,626 controls), AP (294 cases and 104,247 controls), SAB (9,113 cases and 89,340 controls), PROM (3,011 cases and 104,247 controls), GDM (5,687 cases and 117,892 controls), and PE or eclampsia (3,903 cases and 114,735 controls) were obtained from results of the GWAS on the FinnGen consortium R5. Detailed data on the involved cohorts, genotypes, endpoint definition, and association test in the FinnGen consortium are available on the FinnGen webpage.

### Instrumental variable selection

The accuracy of the conclusion on the causal effect of enteric microbiota on APOs was ensured by the following quality control steps used for the section of genetic predictors associated with microbiome features. We selected instrumental variables (IVs) with the locus-wide significance level (*p* < 1 × 10^−5^) to obtain a more comprehensive result (Sanna et al., 2019). The impact of linkage disequilibrium (LD) among included genetic variants on the results was avoided by excluding the SNPs using the PLINK clumping method (r^2^ > 0.001 and clump window <10,000 kb). We then extracted the corresponding data of the selected SNPs from the GWAS outcome data. When SNPs related to exposure were absent in the GWAS outcome data, the proxy SNPs with high LD (r^2^ > 0.80) would be selected. We removed palindromic SNPs to ensure the effects of SNPs on exposure correspond to the same allele as the effects of SNPs on the outcome during the harmonizing process.

### Mendelian randomization analysis

**Figure 2:**
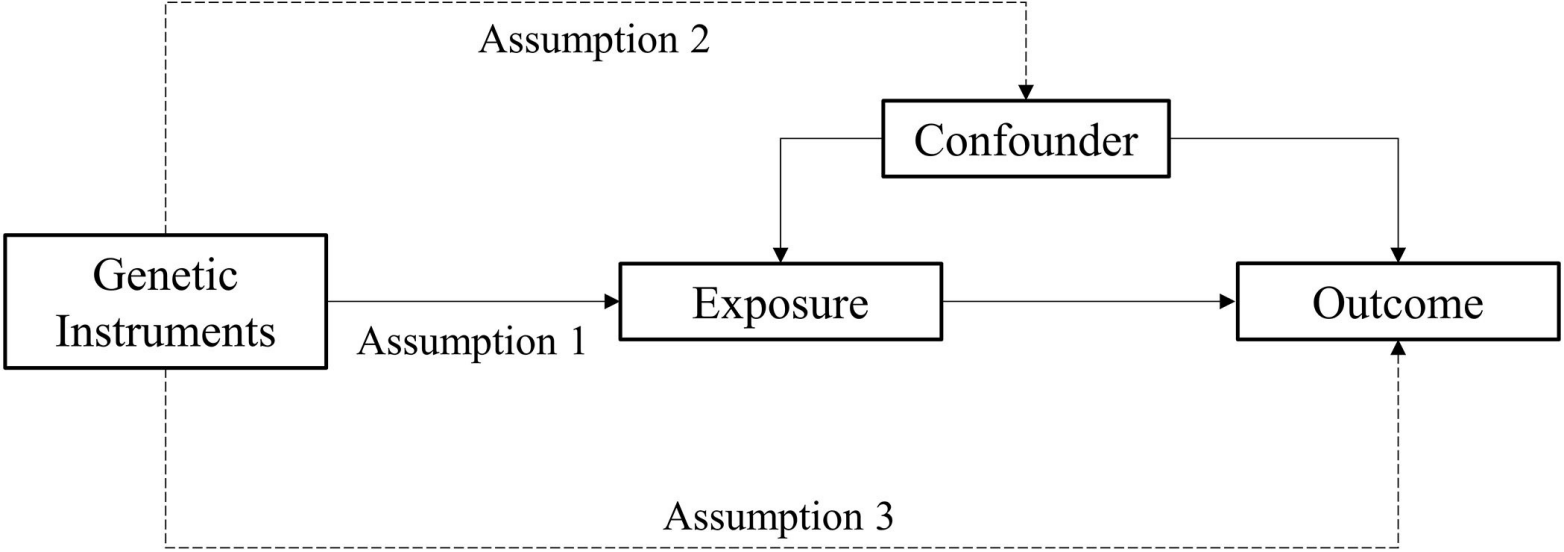
Assumption of the Mendelian randomization study.

MR analysis is a method used to estimate the magnitude of a causal effect of a phenotype on an outcome by using genetic variants such as IVs (Burgess et al., 2011; Davey Smith and Hemani, 2014). There are three important assumptions of MR analysis, which are depicted in Figure 2. The first assumption is that variants should be associated with exposure. The second assumption is that variants should not be associated with any confounders. The third assumption is that the association of genetic variants with outcomes should only be through exposure (Burgess and Thompson, 2015; Yuan S. et al., 2021).

The two-sample IVW method was used as the main MR analysis to estimate the causal relationship of gut microbiota with APOs (Burgess et al., 2013). We performed sensitivity analysis in order to assess the significance of our results. We used the MR-Egger regression and MR-PRESSO to confirm the presence of horizontal pleiotropy (Bowden et al., 2015; Verbanck et al., 2018). Using MR-Egger regression, we can assess whether genetic instruments have pleiotropic effects on the outcome (Bowden et al., 2015). Compared with MR-PRESSO, the MR-Egger method has less precision and statistical power. The MR-PRESSO can detect horizonal pleiotropy and eliminate the effects of pleiotropy by conducting outlier removal (Verbanck et al., 2018). If horizontal pleiotropy was detected among the selected SNPs, the analyses were repeated after removing these pleiotropic SNPs. We performed Cochran's Q test to evaluate the heterogeneity among SNPs associated with each microbial taxon (Egger et al., 1997). The leave-one-out sensitivity analysis was used to assess the effects of a single SNP on the overall estimates. The strength of the selected IVs was assessed using *F* statistics, which determine if estimates of the causal associations are affected by weak instrument bias. We excluded weak IVs with *F* statistics <10 (Pierce et al., 2011). All MR analyses were performed in R (version 4.1.2) using R package TwoSampleMR (Hemani et al., 2018) and MRPRESSO (Verbanck et al., 2018).

## Results

### Selection of instrumental variables

After ensuring quality control, we selected 61 SNPs related to five bacterial taxa for PPH, 109 SNPs related to 10 bacterial taxa for AP, 33 SNPs related to four bacterial taxa for SAB, 81 SNPs related to seven bacterial taxa for PROM, 155 SNPs related to 14 bacterial taxa for GDM, 132 SNPs related to 11 bacterial taxa for PE or eclampsia, and 44 SNPs related to four bacterial taxa for PTB (Supplementary Table S1). The *F* statistics for IVs significantly associated with gut microbiota were all larger than 10, indicating that the estimates were less likely to suffer weak instrument bias.

### Causal effects of gut microbiota on adverse pregnancy outcomes

#### PPH

**Figure 3:**
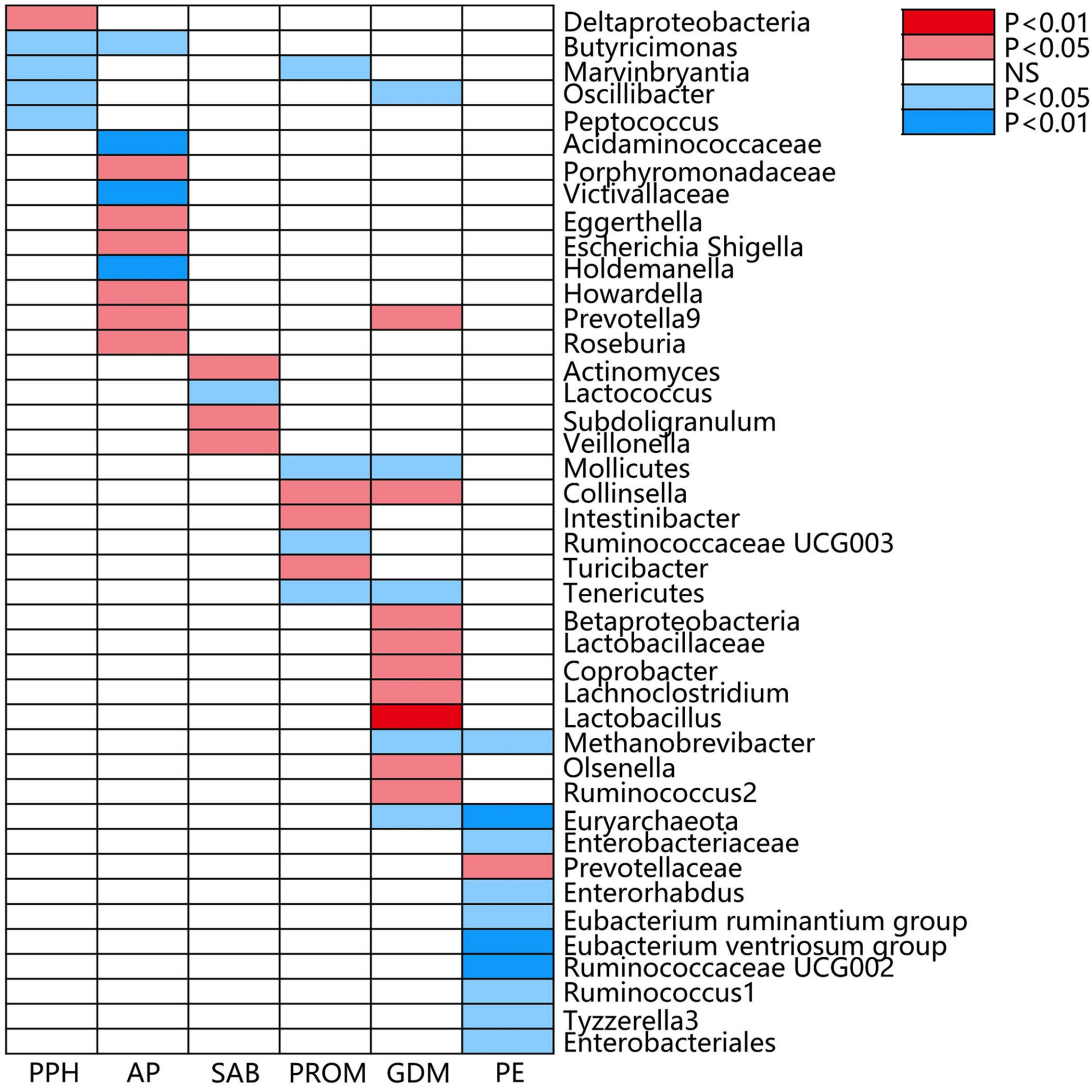
Causal associations of gut microbial genera on adverse pregnancy outcomes (APOs) identified at the nominal significance from the IVW method (*P* < 0.05/0.01). Red represents the risk bacterial traits for APOs, blue represents the protective bacterial traits for APOs, and white represents no causal bacterial traits for APOs. PPH, postpartum hemorrhage; AP, abruptio placentae; SAB, spontaneous abortion; PROM, premature rupture of membrane; GDM, gestational diabetes mellitus; PE, pre-eclampsia; NS, no significant association.

The estimates of the IVW test indicated that the genetically predicted relative abundance of five genera, namely, *Deltaproteobacteria, Butyricimonas, Marvinbryantia, Oscillibacter*, and *Peptococcus*, was associated with an increased or reduced risk of PPH (Table 1; Figure 3) (Chen C. et al., 2020). Specifically, a higher genetically predicted abundance of *Deltaproteobacteria* was associated with a higher risk of PPH (OR: 1.26, 95% CI: 1.00–1.59, *p* = 0.047). A higher genetically predicted abundance of *Butyricimonas* was associated with a reduced risk of PPH (OR: 0.79, 95% CI: 0.63–0.98, *p* = 0.036). The genetically predicted abundance of *Marvinbryantia* was also associated with a reduced risk of PPH (OR: 0.76, 95% CI: 0.59–0.98, *p* = 0.033). The genetically predicted abundance of *Oscillibacter* was negatively related to the risk of PPH (OR: 0.80, 95% CI: 0.64–0.99, *p* = 0.042). The higher genetically predicted abundance of *Peptococcus* was also linked to a reduced risk of PPH (OR: 0.84, 95% CI: 0.72–0.97, *p* = 0.024).

**Table 1:** Causal associations between gut microbiota and the risk of adverse pregnancy complications by using the IVW method.

**Adverse pregnancy complications (outcome)**	**Bacterial taxa (exposure)**	**N**	**OR**	**95% CI**	***P* value**
PPH	Deltaproteobacteria	13	1.26	1.00–1.59	0.047
	Butyricimonas	13	0.79	0.63–0.98	0.036
	Marvinbryantia	10	0.76	0.59–0.98	0.033
	Oscillibacter	13	0.80	0.64–0.99	0.042
	Peptococcus	12	0.84	0.72–0.97	0.024
AP	Acidaminococcaceae	7	0.30	0.12–0.74	0.008
	Porphyromonadaceae	9	3.96	1.24–12.71	0.020
	Victivallaceae	12	0.57	0.37–0.87	0.009
	Butyricimonas	13	0.43	0.20–0.92	0.030
	Eggerthella	10	1.78	1.00–3.17	0.049
	Escherichia Shigella	10	2.73	1.11–6.74	0.029
	Holdemanella	11	0.41	0.23–0.73	0.002
	Howardella	9	1.75	1.10–2.80	0.019
	Prevotella9	15	1.97	1.09–3.57	0.026
	Roseburia	13	3.24	1.29–8.10	0.012
SAB	Actinomyces	7	1.17	1.01–1.36	0.041
	Lactococcus	9	0.90	0.81–1.00	0.041
	Subdoligranulum	11	1.21	1.02–1.43	0.030
	Veillonella	6	1.24	1.03–1.49	0.024
PROM	Mollicutes	12	0.77	0.61–0.98	0.034
	Collinsella	9	1.44	1.03–2.03	0.034
	Intestinibacter	15	1.30	1.05–1.62	0.018
	Marvinbryantia	10	0.74	0.55–0.98	0.034
	Ruminococcaceae UCG003	12	0.73	0.57–0.95	0.017
	Turicibacter	10	1.28	1.02–1.61	0.033
	Tenericutes	12	0.77	0.61–0.98	0.034
GDM	Betaproteobacteria	11	1.26	1.00–1.58	0.047
	Mollicutes	12	0.81	0.67–0.97	0.022
	Lactobacillaceae	8	1.19	1.02–1.39	0.032
	Collinsella	9	1.32	1.01–1.74	0.044
	Coprobacter	10	1.21	1.04–1.41	0.015
	Lachnoclostridium	13	1.37	1.06–1.77	0.017
	Lactobacillus	9	1.24	1.07–1.43	0.004
	Methanobrevibacter	6	0.85	0.73–1.00	0.043
	Olsenella	10	1.17	1.03–1.32	0.016
	Oscillibacter	13	0.82	0.71–0.96	0.011
	Prevotella9	15	1.16	1.01–1.34	0.036
	Ruminococcus2	15	1.19	1.00–1.42	0.047
	Euryarchaeota	12	0.87	0.78–0.97	0.013
	Tenericutes	12	0.81	0.67–0.97	0.022
PE or eclampsia	Enterobacteriaceae	7	0.67	0.49–0.93	0.015
	Prevotellaceae	16	1.24	1.00–1.52	0.049
	Enterorhabdus	6	0.75	0.58–0.97	0.027
	Eubacterium ruminantium group	18	0.87	0.75–1.00	0.049
	Eubacterium ventriosum group	15	0.73	0.59–0.91	0.006
	Methanobrevibacter	6	0.80	0.67–0.97	0.021
	Ruminococcaceae UCG002	22	0.77	0.63–0.93	0.008
	Ruminococcus1	10	0.75	0.56–1.00	0.048
	Tyzzerella3	13	0.83	0.72–0.97	0.016
	Enterobacteriales	7	0.67	0.49–0.93	0.015
	Euryarchaeota	12	0.81	0.72–0.92	0.001

#### AP

The estimates of the IVW test suggested that the genetically predicted relative abundance of four genera, namely, *Acidaminococcaceae* (OR: 0.30, 95% CI: 0.12–0.74, *p* = 0.008), *Victivallaceae* (OR: 0.57, 95% CI: 0.37–0.87, *p* = 0.009), *Butyricimonas* (OR: 0.43, 95% CI: 0.20–0.92, *p* = 0.030), and *Holdemanella* (OR: 0.41, 95% CI: 0.23–0.73, *p* = 0.002), was negatively associated with the risk of AP. The genetically predicted relative abundance of a different five genera, namely, *Porphyromonadaceae* (OR: 3.96, 95% CI: 1.24–12.71, *p* = 0.020), *Eggerthella* (OR: 1.78, 95% CI: 1.00–3.17, *p* = 0.049), *Escherichia Shigella* (OR: 2.73, 95% CI: 1.11–6.74, *p* = 0.029), *Howardella* (OR: 1.75, 95% CI: 1.10–2.80, *p* = 0.019), *Prevotella9* (OR: 1.97, 95% CI: 1.09–3.57, *p* = 0.026), and *Roseburia* (OR: 3.24, 95% CI: 1.29–8.10, *p* = 0.012), was positively associated with the risk of AP.

#### SAB

A higher genetically predicted abundance of *Actinomyces* (OR: 1.17, 95% CI: 1.01–1.36, *p* = 0.041), *Subdoligranulum* (OR: 1.21, 95% CI: 1.02–1.43, *p* = 0.030), and *Veillonella* (OR: 1.24, 95% CI: 1.03–1.49, *p* = 0.024) was significantly associated with the risk of SAB. There was a tendency toward a protective effect of increasing genetically predicted abundance of *Lactococcus* on the risk of SAB (OR: 0.90, 95% CI: 0.81–1.00, *p* = 0.041).

#### PROM

The genetically predicted relative abundance of three genera, namely, *Collinsella* (OR: 1.44, 95% CI: 1.03–2.03, *p* = 0.034), *Intestinibacter* (OR: 1.30, 95% CI: 1.05–1.62, *p* = 0.018), and *Turicibacter* (OR: 1.28, 95% CI: 1.02–1.61, *p* = 0.033), was associated with an increased risk of PROM. By contrast, there was a tendency toward a protective effect of increasing genetically predicted abundance of *Mollicutes* (OR: 0.77, 95% CI: 0.61–0.98, *p* = 0.034), *Marvinbryantia* (OR: 0.74, 95% CI: 0.55–0.98, *p* = 0.034), *Ruminococcaceae UCG003* (OR: 0.73, 95% CI: 0.57–0.95, *p* = 0.017), and *Tenericutes* (OR: 0.77, 95% CI: 0.61–0.98, *p* = 0.034) on the risk of PROM.

#### GDM

The estimates of the IVW test suggested that the genetically predicted relative abundance of five genera, namely, *Mollicutes* (OR: 0.81, 95% CI: 0.67–0.97, *p* = 0.022), *Methanobrevibacter* (OR: 0.85, 95% CI: 0.73–1.00, *p* = 0.043), *Oscillibacter* (OR: 0.82, 95% CI: 0.71–0.96, *p* = 0.011), *Euryarchaeota* (OR: 0.87, 95% CI: 0.78–0.97, *p* = 0.013), and *Tenericutes* (OR: 0.81, 95% CI: 0.67–0.97, *p* = 0.022), was in negatively associated with the risk of GDM, whereas the genetically predicted relative abundance of nine other genera, namely, *Betaproteobacteria* (OR: 1.26, 95% CI: 1.00–1.58, *p* = 0.047), *Lactobacillaceae* (OR: 1.19, 95% CI: 1.02–1.39, *p* = 0.032), *Collinsella* (OR: 1.32, 95% CI: 1.01–1.74, *p* = 0.044), *Coprobacter* (OR: 1.21, 95% CI: 1.04–1.41, *p* = 0.015), *Lachnoclostridium* (OR: 1.37, 95% CI: 1.06–1.77, *p* = 0.017), *Lactobacillus* (OR: 1.24, 95% CI: 1.07–1.43, *p* = 0.004), *Olsenella* (OR: 1.17, 95% CI: 1.03–1.32, *p* = 0.016), *Prevotella9* (OR: 1.16, 95% CI: 1.01–1.34, *p* = 0.036), and *Ruminococcus2* (OR: 1.19, 95% CI: 1.00–1.42, *p* = 0.047), was positively associated with the risk of GDM.

#### PE or eclampsia

The genetically predicted abundance of *Prevotellaceae* (OR: 1.24, 95% CI: 1.00–1.52, *p* = 0.049) was associated with an increased risk of PE or eclampsia. By contrast, there was a tendency toward a protective effect of increasing genetically predicted abundance of *Enterobacteriaceae* (OR: 0.67, 95% CI: 0.49–0.93, *p* = 0.015), *Enterorhabdus* (OR: 0.75, 95% CI: 0.58–0.97, *p* = 0.027), *Eubacterium ruminantium* (OR: 0.87, 95% CI: 0.75–1.00, *p* = 0.049), *Eubacterium ventriosum* (OR: 0.73, 95% CI: 0.59–0.91, *p* = 0.006), *Ruminococcaceae UCG002* (OR: 0.77, 95% CI: 0.63–0.93, *p* = 0.008), *Ruminococcus1* (OR: 0.75, 95% CI: 0.56–1.00, *p* = 0.048), *Tyzzerella3* (OR: 0.83, 95% CI: 0.72–0.97, *p* = 0.016), *Enterobacterales* (OR: 0.67, 95% CI: 0.49–0.93, *p* = 0.015), and *Euryarchaeota* (OR: 0.81, 95% CI: 0.72–0.92, *p* = 0.001) on PE or eclampsia.

### Sensitivity analysis

We did not find pleiotropic effects among the selected SNPs using the MR-Egger and MR-PROSSO global test (*p* > 0.05) (Supplementary Tables S2, S3). No evidence of heterogeneity was observed between the selected IVs and APOs (Supplementary Table S4). In addition, we found no causal relationships between identified bacterial taxa and APOs that were driven by any single SNP (Supplementary Table S5).

## Discussion

In the present study, we found causal relationships between genetically predicted abundance of specific bacterial taxa and six common pregnancy complications (PPH, AP, SAB, PROM, GDM, and PE or eclampsia) using large-scale GWAS summary data.

Considerable interest in the potential molecular mechanisms of the gut microbiome in the pathogenesis of PPH was evidenced by an abundance of prior studies. The gut microbiome composition was found to be involved in the pathogenesis of PPH by regulating inflammation, which are closely associated with development of PPH (Farhana et al., 2015; Yang et al., 2021). We successfully identified that *Butyricimonas, Marvinbryantia, Oscillibacter*, and *Peptococcus* were negatively associated with the risk of PPH. *Butyricimonas, Marvinbryantia*, and *Oscillibacter* can produce butyrate, which participates in regulating gut permeability and induces the inflammatory response (Jang et al., 2020; Chen B. D. et al., 2021; Chen Z. et al., 2021; Liu et al., 2022). Prior studies have reported that the abundance of *Peptococcus* increases when intestinal inflammation occurs, indicating its pro-inflammatory effects (Sun et al., 2022). The exact mechanism of *Peptococcus* on the pathogenesis of PPH warrants verification studies. This study also suggested that increased abundance of *Deltaproteobacteria* was causally related to a higher risk of PPH. *Deltaproteobacteria* is a common pathogenic bacterium, which initiates inflammation by activating the Notch signaling pathway and induces the expression of pro-inflammatory factors including pro-IL-1β and SOCS3 (Jin et al., 2016; Singh et al., 2021).

Emerging evidence has shown the role of perturbations in mitochondrial biogenesis, oxidative phosphorylation, and oxidative stress pathways in the pathogenesis of AP (Workalemahu et al., 2018). The relationship between gut microbiota dysbiosis and AP remains unclear. This study found that the abundance of *Butyricimonas* and *Holdemanella* reduced the risk of AP. The association may be mediated by the short-chain fatty acid butyrate as *Butyricimonas* and *Holdemanella* are involved in its production (Zagato et al., 2020; Jing et al., 2021). Butyrate has a key role in maintaining mucosal integrity, reducing inflammation *via* macrophage function, and regulating oxidative stress (Hu et al., 2020; Jing et al., 2021). We also showed that abundance of *Eggerthella* and *Escherichia Shigella* was positively associated with the risk of AP. *Eggerthella* is a pro-inflammatory genus, which has been associated with the depletion of butyrate (Nikolova et al., 2021). *Escherichia Shigella* can also mediate inflammation and oxidative stress by regulating the lipopolysaccharide/TLR4/NF-kB signaling pathway (Peng et al., 2020). This study demonstrated that *Roseburia* and *Porphyromonadaceae*, butyrate-producing bacteria, were positively related to the risk of AP (Wang et al., 2022). It can suggest that a single microbial taxon may not affect the development of AP, and the overall role of gut microbiota may have a more important role in the onset of AP. We showed that some novel gut bacteria including *Acidaminococcaceae, Victivallaceae, Howardella*, and *Prevotella9* were closely associated with the pathogenesis of AP, but the exact mechanism of these gut microbiome on the development of AP warrants additional investigations.

We discovered that the abundance of *Actinomyces, Subdoligranulum*, and *Veillonella* was associated with an increased risk of SAB, while a high abundance of *Lactococcus* was related to a lower risk of SAB. Immunologic dysfunction and thrombophilia are known key factors in the pathogenesis of SAB (Liu et al., 2021a). A previous study showed that the relative abundance of *Actinomyces* and *Subdoligranulum* is closely related to multiple autoimmune diseases, including systemic lupus erythematosus and chronic spontaneous urticaria, suggesting that these bacteria play an important role in the host immune response (Chen B. D. et al., 2021; Liu et al., 2021). The protective effect of *Lactococcus* on SAB was likely due to lactic acid bacteria reducing autoimmunity and recovering immune homeostasis (Xu et al., 2020). Increased thrombophilia is one of the clinical signs of SAB (de Jong et al., 2014). *Veillonella* may influence the host trimethylamine N-oxide (TMAO) level by mediating carnitine production and metabolism, which contribute to platelet hyperreactivity and thrombosis (Yang et al., 2019). The exact the mechanism of gut microbiota leading to SAB remains poorly understood and warrants additional studies.

Although exact mechanisms underlying the occurrence of PROM are not fully elucidated, oxidative stress and inflammation have been reported to play an important role (Liu et al., 2021b). Our study revealed that the genetically predicted abundance of *Turicibacter* was positively correlated to the risk of PROM. Animal studies have reported that the abundance of *Turicibacter* was negatively associated with propionic acid and butyric acid, which can inhibit the inflammatory response (Wan et al., 2021). We have found that an increased abundance of *Marvinbryantia* and *Ruminococcaceae*, which are known to produce short-chain fatty acid, was negatively associated with the risk of PROM (Chen B. D. et al., 2021; Chen Z. et al., 2021).

Previous studies on GDM or post-GDM women with impaired glucose tolerance discovered a broad range of gut microbiota dysbiosis, which was associated with *Collinsella* (Crusell et al., 2018), *Olsenella* (Crusell et al., 2018), *Prevotella9* (Hasain et al., 2020), and *Ruminococcus2* (Crusell et al., 2018). On the other hand, beneficial butyrate-producing bacteria including *Methanobrevibacter* (Kuang et al., 2017) and *Oscillibacter* (Crusell et al., 2018) were depleted. A prior study also indicated that individuals with diminished insulin sensitivity had a lower abundance of *Tenericutes*, suggesting it might be associated with the occurrence and development of GDM (Yuan X. et al., 2021). Larsen et al. found that the relative abundance of *Betaproteobacteria* was higher in the type 2 diabetes group and positively correlated with the plasma glucose level (Larsen et al., 2010). These results were consistent with the present study. The mechanisms through which these bacteria exert beneficial or detrimental effects on the pathogenesis of GDM deserve to be studied.

Our study also showed that the abundance of *Eubacterium ruminantium, Eubacterium ventriosum, Ruminococcaceae UCG002, Ruminococcus1*, and *Enterobacterales* was negatively linked to the risk of PE or eclampsia. Prior studies indicated that chronic peripheral and placental inflammation were critical to the onset of PE (Black and Horowitz, 2018). *Eubacterium, Ruminococcaceae*, and *Enterobacterales* are involved in the production of short-chain fatty acids, such that their depletion is causally linked to inflammation (Tran et al., 2019; Zhuang et al., 2020). Wang et al. (2019) showed that fecal microbiota showed a significant reduction of *Ruminococcus* in the PE group, which was important in maintaining gut homeostasis. This study also demonstrated that some bacteria such as *Prevotellaceae, Enterobacteriaceae, Enterorhabdus*, and *Euryarchaeota* were involved in the pathogenesis of PE. Future studies are warranted to elucidate the functional significance of these bacteria in the onset of PE.

The advantages of this study as follows: First, the MR design diminishes the residual confounding and reverse causality and thereby improves the causal inference in relationships of gut microbiota and APOs. Second, we comprehensively studied up to six common APOs. Third, the identified causal associations in our MR analysis may provide candidate microbial taxa for subsequent functional studies and help in the development of new approaches for the prevention and treatment of pregnancy complications by targeting specific gut bacteria.

The limitations of our study must also be acknowledged. First, SNPs obtained based on the genome-wide statistical significance threshold (5 × 10^−8^) were too limited for further study; therefore, we included the SNPs that met the locus-wide significance level (1 × 10^−5^) in this study. Second, while the GWAS summary data in this study were obtained mostly from European populations, only a small amount of the gut microbiome data was taken from other races, which may partially bias our findings. Third, we cannot exclude the possible diet–gene or gene–environment interactions on outcomes, which may influence the observed results.

In conclusion, this MR study comprehensively explores causal effects of gut microbiota on APOs for the first time. Our findings provide new insights into the prevention, course of disease, and treatment of APOs by targeting specific bacteria taxa. Further studies are warranted to identify the exact mechanism of enteric microbiota–pregnancy complication associations.

## Data availability statement

Summary data used for this study can be accessed through the following links: microbiota, https://mibiogen.gcc.rug.nl/; post-partum hemorrhage, https://r5.finngen.fi/pheno/O15_POSTPART_HEAMORRH; abruptio placenta, https://r5.finngen.fi/pheno/O15_PLAC_PREMAT_SEPAR; spontaneous abortion, https://r5.finngen.fi/pheno/O15_ABORT_SPONTAN; premature rupture of membrane, https://r5.finngen.fi/pheno/O15_MEMBR_PREMAT_RUPT; gestational diabetes mellitus, https://r5.finngen.fi/pheno/GEST_DIABETES; pre-eclampsia or eclampsia, https://r5.finngen.fi/pheno/O15_PRE_OR_ECLAMPSIA.

## Ethics statement

This study was approved by Ethics Committee of Shengjing Hospital of China Medical University.

## Author contributions

CLi and CLiu designed the research. CLi collected and analyzed the data and drafted the manuscript. CLiu and NL supervised the study. CLi, CLiu, and NL were involved in writing the manuscript. All authors contributed to the article and approved the submitted version.
